# Lipid emulsion interfacial design modulates human *in vivo* digestion and satiation hormone response

**DOI:** 10.1039/d2fo01247b

**Published:** 2022-08-02

**Authors:** Pascal Bertsch, Andreas Steingoetter, Myrtha Arnold, Nathalie Scheuble, Jotam Bergfreund, Shahana Fedele, Dian Liu, Helen L. Parker, Wolfgang Langhans, Jens F. Rehfeld, Peter Fischer

**Affiliations:** Laboratory of Food Process Engineering, Department of Health Sciences and Technology, Institute of Food Nutrition and Health, ETH Zurich Zurich Switzerland peter.fischer@hest.ethz.ch; Division of Gastroenterology and Hepatology, University Hospital Zurich Zurich Switzerland; Department of Information Technology and Electrical Engineering, Institute for Biomedical Engineering, University and ETH Zurich Zurich Switzerland; Physiology and Behavior Laboratory, Department of Health Sciences and Technology, Institute of Food Nutrition and Health, ETH Zurich Zurich Switzerland; Northern Medical Physics and Clinical Engineering, Royal Victoria Infirmary, Newcastle upon Tyne NHS Trust Hospitals Newcastle upon Tyne UK; Department of Clinical Biochemistry, Rigshospitalet, University of Copenhagen Copenhagen Denmark

## Abstract

Lipid emulsions (LEs) with tailored digestibility have the potential to modulate satiation or act as delivery systems for lipophilic nutrients and drugs. The digestion of LEs is governed by their interfacial emulsifier layer which determines their gastric structuring and accessibility for lipases. A plethora of LEs that potentially modulate digestion have been proposed in recent years, however, *in vivo* validations of altered LE digestion remain scarce. Here, we report on the *in vivo* digestion and satiation of three novel LEs stabilized by whey protein isolate (WPI), thermo-gelling methylcellulose (MC), or cellulose nanocrystals (CNCs) in comparison to an extensively studied surfactant-stabilized LE. LE digestion and satiation were determined in terms of gastric emptying, postprandial plasma hormone and metabolite levels characteristic for lipid digestion, perceived hunger/fullness sensations, and postprandial food intake. No major variations in gastric fat emptying were observed despite distinct gastric structuring of the LEs. The plasma satiation hormone and metabolite response was fastest and highest for WPI-stabilized LEs, indicating a limited capability of proteins to prevent lipolysis due to fast hydrolysis under gastric conditions and displacement by lipases. MC-stabilized LEs show a similar gastric structuring as surfactant-stabilized LEs but slightly reduced hormone and metabolite responses, suggesting that thermo-gelling MC prevents lipase adsorption more effectively. Ultimately, CNC-stabilized LEs showed a drastic reduction (>70%) in plasma hormone and metabolite responses. This confirms the efficiency of particle (Pickering) stabilized LEs to prevent lipolysis proposed in literature based on *in vitro* experiments. Subjects reported more hunger and less fullness after consumption of LEs stabilized with MC and CNCs which were able to limit satiation responses. We do not find evidence for the widely postulated ileal brake, *i.e.* that delivery of undigested nutrients to the ileum triggers increased satiation. On the contrary, we find decreased satiation for LEs that are able to delay lipolysis. No differences in food intake were observed 5 h after LE consumption. In conclusion, LE interfacial design modulates *in vivo* digestion and satiation response in humans. In particular, Pickering LEs show extraordinary capability to prevent lipolysis and qualify as oral delivery systems for lipophilic nutrients and drugs.

## Abstract 2

Lipid emulsions (LEs) with tailored digestibility have the potential to modulate satiation or act as delivery systems for lipophilic nutrients and drugs.

## Graphical abstract

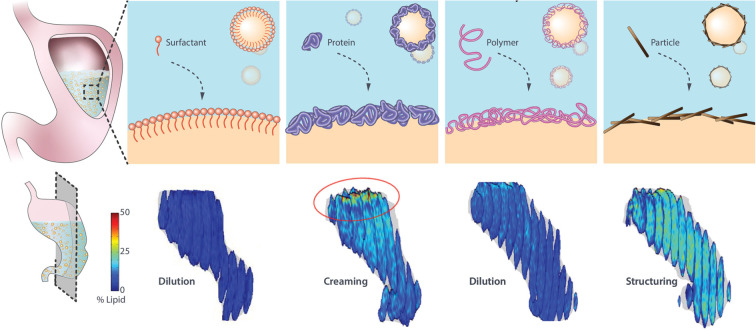

## 1. Introduction

Lipids are an essential part of the human diet, however, as the most energy-dense macronutrients they are a leading contributor to caloric over-consumption associated with obesity, diabetes, and coronary diseases. Many dietary lipids are ingested in form of lipid emulsions (LEs), *i.e.* micron-sized oil droplets dispersed in continuous water (also called oil-in-water (o/w) emulsions). The rate at which LEs are digested and induce a satiation response is determined by their structuring and stability under gastric conditions. This has been recognized as promising approach to design LEs with the ability to modulate satiation or delivery of lipophilic pharmaceuticals.^1–7^ The structure and stability of LEs under gastric conditions is mostly determined by the used emulsifier. Emulsifiers are amphiphilic molecules that assemble at the o/w interface and increase emulsion stability by a decrease in surface tension (thermodynamic stability) and/or steric or electrostatic repulsion (kinetic stability). Commonly used emulsifiers comprise small molecular weight surfactants, proteins, and more recently also solid particles (Pickering stabilization).^6–10^ The interfacial emulsifier layer determines the digestion of LEs by two distinct mechanisms. First, the type of emulsifier affects the structuring and stability of LEs under gastric conditions.^1,11^ Magnetic resonance imaging (MRI) revealed that surfactant-stabilized LEs tend to be well distributed in the stomach, while protein-stabilized LEs rapidly coalesce and cream due to protease activity, and LEs stabilized by charged particles form gel-like structures that impede the diffusion of gastric enzymes.^7,12–14^ Furthermore, gastric unstable LEs invoke an increase in emulsion droplet size which reduces the specific surface area for lipolysis, *i.e.* the cleavage of triglycerides (TAGs) into absorbable monoglycerides (MAGs) and free fatty acids (FFAs).^15,16^ Second, in order for lypolysis to occur the lipases have to replace the existing emulsifier layer.^17,18^ For surfactants, the ability to prevent lipase adsorption is determined by the surfactant affinity towards the oil phase and headgroup charge.^9^ Proteins are generally rapidly displaced by lipases or hydrolyzed by proteases.^19,20^ On the other hand, solid particles have been reported to effectively prevent lipolysis.^6,7,10,21^ Collectively these two distinct mechanisms determine the digestibility of LEs which ultimately regulates satiation, as absorption of MAGs and FFAs in the duodenum trigger the release of gastrointestinal (GI) satiation hormones. GI hormones commonly associated with lipid absorption are cholecystokinin (CCK), peptide YY (PYY), and glucagon-like peptide 1 (GLP-1).^16,22^

**Fig. 1:**
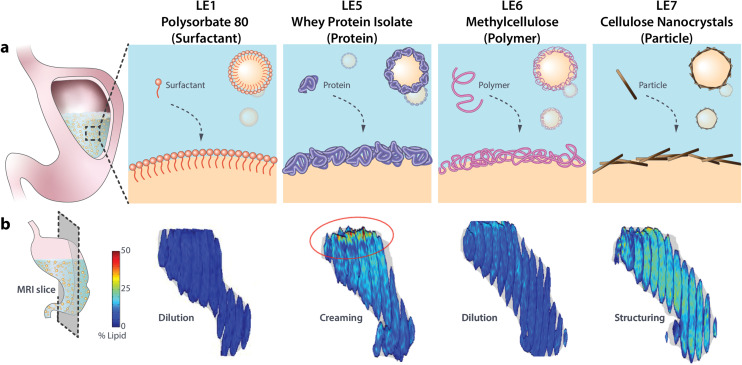
(a) Schematic of different LE interfacial designs and (b) magnetic resonance images showing their *in vivo* gastric structuring and lipid distribution. Magnetic resonance images are reproduced with permission from Liu *et al.*^12^ (PS-LE1) and Scheuble *et al.*^7^ (WPI-LE5, MC-LE6, CNC-LE7).

Many LE systems have been designed in recent years with the potential to modulate satiation or deliver pharmaceuticals. The gastric structuring and rate of lipolysis of LEs is commonly determined by *in vitro* digestion models, mostly using the INFOGEST protocol.^23^ However, LE digestion can differ considerably *in vivo* particularly when regarding gastric structuring^7^ and LE-specific emptying rates.^24^ Hence, *in vivo* validation of such LEs and their effect on satiation are crucial, but remain scarce to date. Clifton and colleagues^25–27^ introduced a set of emulsions with altering emulsifier type to study their effects on *in vivo* gastric structuring, fat emptying, and satiation hormone response. These LEs, which were serially numbered LE1–LE4, have since been readily employed for *in vivo* experiments.^12,24,28–30^ We have recently expanded this set by three novel LEs (termed LE5–LE7 for consistency) stabilized with alternative emulsifiers which are able to alter gastric structuring and lipolysis in humans.^7^ Here, we further assess the ability of LE5–LE7 to modulate *in vivo* digestion in terms of gastric emptying, plasma metabolite and hormone response, postprandial hunger and food consumption in humans compared to the established LE1. An overview of the interfacial design and gastric structuring of the four tested LEs is provided in Fig. 1.

LE1 is the most extensively studied LE and serves here as a reference for the newly designed LE5–LE7. LE1 is stabilized by the small molecular weight surfactant polysorbate 80. LE1 is stable and evenly dispersed in the stomach, exhibits a steady gastric emptying, and relatively high levels of plasma FFA and satiation hormones compared to other LEs.^24,25,27–30^ LE5 is stabilized by whey protein isolate (WPI), a common emulsifier in food industry. As is typical for protein-stabilized LEs, LE5 is unstable under gastric conditions due to rapid displacement by lipases and proteolysis leading to lipid layering in the stomach.^7,12–14,28^ LE6 is stabilized by thermo-gelling methylcellulose. The methylation renders the cellulose surface-active for efficient emulsion stabilization and thermo-gelling at human body temperature which enhances interfacial layer thickness and can prevent lipase adsorption.^31–33^ Finally, LE7 is stabilized by nanoparticles (cellulose nanocrystals (CNCs)) to account for the increasing use of Pickering emulsions. CNCs are charged anisotropic nanoparticles that form dense interfacial particle layers upon emulsification.^34^ In our previous study LE7 was shown to induce strong gastric gelling, impede lipase adsorption, and induce very limited hormone response in healthy humans.^7^ The LEs are henceforward termed using both their specific emulsifier and number identifier, *i.e.* PS-LE1, WPI-LE5, MC-LE6, and CNC-LE7.

## 2. Study design, materials and methods

### 2.1. Emulsion design

LEs were produced from 20 wt% canola oil (Sabo) and Evian water using different emulsifiers. PS-LE1 was prepared according to previous studies^12,24,25,27–29^ using 0.8 wt% small molecular weight surfactant polysorbate 80 (Palsgaard) and 0.8 wt% xanthan (KELTROL, CP Kelco) to increase viscosity. The novel LE5–LE7 were prepared as described in detail by Scheuble *et al.*^7^ In brief, WPI-LE5 was stabilized by 1 wt% WPI (BiPro, Davisco), MC-LE6 was stabilized by 4 wt% MC A15 (Dow Chemicals), and CNC-LE7 was stabilized by 4 wt% CNCs (CelluForce). The emulsifiers were dispersed in the aqueous phase under stirring for 1 h (WPI), 2 h (CNCs) or overnight at 4 °C (MC). The oil was added and pre-emulsions were formed using a rotor-stator blender before emulsifying using a high-pressure Microfluidizer M-110EH-30 (Microfluidics Corp.). Emulsions were pasteurized for 5 min at 75 °C. The droplet diameter was ≈0.3 μm for PS-LE1, 0.4 μm for MC-LE6, and 1 μm for WPI-LE5 and CNC-LE7 as reported in our previous studies.^7,24^ For LEs stabilized with the same emulsifier a smaller droplet size results in a higher specific surface area for lipolysis which can accelerate satiation hormone response.^16^ However, for LEs stabilized with different emulsifiers, emulsifier-specific effects like gastric structuring, changes in droplet size during digestion, and interfacial competition with lipases are more pronounced.^7^ The complex viscosity of the LEs was ≈0.2 Pa s for WPI-LE5 and MC-LE6 and ≈20 Pa s for CNC-LE7.^7^

### 2.2. Extensional rheometry

Capillary breakup extensional rheometry (CaBER) measurements were carried out on a CaBER 1 (Thermo Fisher Scientific) to estimate flow properties of the LEs during emptying. The sample was loaded between the two plates, which were separated with an initial step strain (50 ms). A liquid bridge was formed between the two cylindrical test fixtures followed by a self-driven uniaxial extensional flow, which leads to a breakup of the filament. The response of a fluid following an axial step-strain is encoded in an apparent transient elongational viscosity function, which can be determined by measuring the change of the filament diameter *D*_mid_ and strain rate 
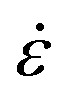
 as a function of time. The resulting system Hencky strain *ε* is defined as 
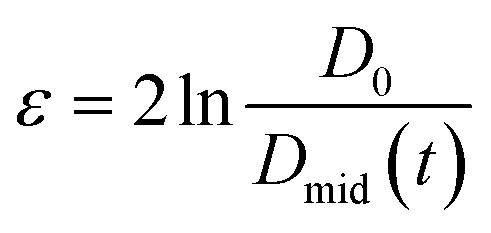
 with *D*_0_ being the initial diameter of the fluid thread before stretching.^35^ The measurements were performed at an initial gap of 3 mm and a sample final height of 12.03 mm with an initial aspect ratio of 1 and a final aspect ratio of 4.01. The measurements were conducted at room temperature.

### 2.3. Overview of human study design

**Fig. 2:**
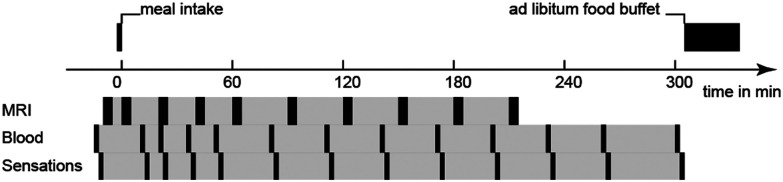
Study day timeline in the human study.

The human study was approved by the local ethics committee and was registered at ClinicalTrials.gov with identifier NCT02865486. The study was a randomized, double-blinded, unbalanced three-way crossover study. Eligible healthy subjects had a body mass index of 18–25 kg m^−2^, no current health problems or past GI disease, no history of abdominal surgery (excluding appendectomy or hernia repair), were non-smokers and not pregnant. 21 subjects were recruited and randomly assigned to three of four LEs on three different study days. Four subjects had to be excluded: one subject was unable to attend, two subjects suffered from nausea and one subject could not be cannulated. The final subject number per LE and power consideration are indicated in the statistical analysis section. On each study day the subjects arrived fasted. They underwent baseline MRI scans, blood sampling and rated their subjective sensation of hunger and fullness before ingesting LEs. After LE ingestion MRI scans, blood sampling, and sensations ratings were recorded periodically. Subjects were provided with an *ad libitum* buffet 5 h after LE ingestion, *i.e.* subjects were allowed to serve their own meal size and composition which was weighed before and after consumption, to study postprandial food intake. An overview of the study day timeline is provided in Fig. 2.

#### MRI measurements, blood sampling, and sensations

All MR images were acquired in the right decubitus position. During scan pauses, subjects were allowed to assume a sitting position to ensure that intragastric gas accumulated in the fundus and was excluded from the antrum.^36^ 200 mL (380 kcal) of each LE was ingested within 1–2 min in a seated position. Postprandial MRI was performed in nine scan blocks at time points *t* = 0 (end of ingestion), 20, 40, 60, 90, 120, 150, 180 and 210 min. In each scan block, images were acquired to assess gastric and gallbladder content volumes (GV). Two venous blood samples were taken in fasting state (baseline) and thereafter at time points *t* = 10, 20, 35, 50, 80, 110, 140, 170, 200, 250 and 300 min. Plasma levels of the GI hormones GLP-1 and PYY were determined by chemiluminescence assays (MESO Scale Discovery). CCK concentrations were determined using a radioimmunoassay with high sensitivity and specificity.^37^ The metabolites TAG, betahydroxybutyrate (BHB), free glycerine (FGY) and glucose (GLU) were extracted from plasma samples by enzymatic tests adapted for the Cobas Mira autoanalyzer (Hoffman La-Roche). After each blood sampling, subjects rated their subjective sensations of hunger, fullness, nausea, bloating, and epigastric pain. A visual analogue scale from 0 mm (no symptoms) to 100 mm (extremely prominent symptoms) was used for sensation ratings. The weight and selection of foods obtained at the *ad libitum* food buffet was recorded and their respective amount of calories and nutrients was calculated.

### 2.4. Data analysis

#### Gastric emptying

All quantitative image processing was performed under blinded condition. A custom-made software tool written in MATLAB (version R2015a; The MathWorks) was used for a semi-automated image segmentation and quantification of gastric content and gallbladder volumes according to previously reported procedures.^12,29^ Fat volume (FV), secretion volume (SV) and gallbladder volume (GV) were calculated at each time point and plotted over time to generate volume emptying curves.^28^ FV and SV curves were mathematically described with the LinExp model^38^ according to:1
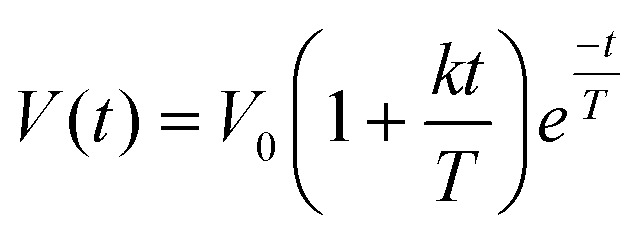
with the three fitted volume parameters for the initial postprandial volume *V*_0_, the initial volume increase or delay in volume decrease *k* and the subsequent volume emptying time *T*.

#### Plasma GI hormones and metabolites

Postprandial delta over baseline (DOB) curves were calculated from the plasma concentrations of the GI hormones CCK, GLP-1, PYY and the metabolites TAG, BHB, FGY, and GLU. The area over baseline AOB, the time-to-maximum/minimum amplitude *t*_max_ and the maximum/minimum amplitude *A*_max_ for each GI hormone or metabolite were computed from the DOB curves using a previously developed power-exponential model^30^ according to:

2 *C*(*t*) = *AOBkβ*(1 − *e*^*kt*^)^*β*−1^*e*^−*kt*^

with *t*_max_ = log *β*/*k* and *A*_max_ = *Ct*_max_. For visceral sensations and food consumption, postprandial DOB curves were calculated from the visceral sensation ratings of hunger, fullness, nausea, bloating, and epigastric pain using the same power-exponential model according to eqn (2).

#### Statistical analysis

Statistical analyses were carried out with R (Version 3.1.3). LinExp and power-exponential model parameters were fitted to the time series data in a hierarchical Bayesian Markov chain Monte Carlo sampling strategy using R package rstan (Version 2.8.2). The effects of the four emulsions on the model parameters were presented as medians and 95% highest posterior density intervals (HPDs). The effect of the four emulsions on food consumption from the *ad libitum* buffet was tested by linear modeling with emulsion, sex, buffet time and the emulsion-sex-interaction as fixed effects. The function lm was used for the linear models and the resulting estimates were presented as means with 95% CI.

The 21 subjects were randomly assigned to 3 out of 4 LEs, of which 17 subjects completed the study. Power considerations based on our previous study^7^ suggested that the least difference is expected between PS-LE1 and MC-LE6, wherefore *n* = 15 would allow for the detection of a difference of 1 ml h^−1^ in the fat emptying rate with a power of 80–90% at a significance level of 1%. Due to strong *in vivo* effects *n* = 10 was sufficient for CNC-LE7. The final subject numbers *n* per LE were PS-LE1 = 14, WPI-LE5 = 14, MC-LE6 = 13, CNC-LE7 = 10. Volume emptying data from four scan blocks and blood data from three samples were missing due to technical errors. Sensations data from two ratings were missing in error. The few missing data points still allowed a robust computation of the gastric and fat emptying as well as hormone/metabolite concentration curves.

## 3. Results

In total 21 subjects participated in the study. Four subjects had to be excluded as indicated in above. 11 female and 6 male subjects with a mean ± SD age of 25.6 ± 5.0 year and a mean ± SD BMI of 22.2 ± 1.8 kg m^−2^ completed the study. Statistical analyses were performed on 455 observations for fitting volume curves, 609 observations for fitting plasma hormone/metabolite concentration curves and 610 observations for fitting sensation curves. Food intake was analyzed based on 51 observations. Table 1 displays all parameter estimates and differences between LEs where the 95% HPDs do not cross zero.

**Table 1:** Estimates of volume, GI hormone, metabolite and sensation parameters and corresponding comparisons for PS-LE1, WPI-LE5, MC-LE6 and CNC-LE7 in healthy subjects. Only differences where the HPD 95% CI does not cross zero are shown (*n* = 51)

Measure	Parameter	Estimate^a^		Difference^b^	
		LE	Value	LE	Value
**Volume**					
Fat	*v*_0_, mL	1	35.3 (33.8, 36.5)	6	−1.8 (−3.2, −0.2)
Secretion	*T*, min	5	112 (82, 139)	6	55 (21, 90)
		6	166 (131, 197)	7	−41 (−85, −10)
	*k*	5	2.6 (1.7, 3.3)	6	1.1 (0.2, 2.0)
Gallbladder	AOB, mL min	1	−245 (−300, −189)	5	66 (0, 127)
	*A*_max_, mL	5	−0.5 (−0.7, −0.4)	7	0.1 (0.0, 0.3)
	*t*_max_, min	5	248 (88, 387)	7	98 (37, 187)
		6	187 (112, 308)	7	82 (25, 174)
**GI hormone**					
Cholecystokinin	AOB, pmol L^−1^ min^−1^	1	76 (54, 92)	5	−37 (−55, −20)
		5	38 (26, 53)	6	31 (15, 56)
	*A*_max_, pmol L^−1^	1	0.13 (0.09, 0.19)	7	−0.09 (−0.14, −0.05)
		5	0.14 (0.09, 0.20)	7	−0.10 (−0.17, −0.05)
		6	0.12 (0.07, 0.16)	7	0.08 (0.03, 0.13)
	*t*_max_, min	5	26 (11, 47)	6	47 (9, 94)
				7	158 (24, 362)
Glucagon-like peptide 1	AOB, pg L^−1^ min^−1^	5	31 (22, 39)	6	16 (4, 33)
	*A*_max_, pg mL^−1^	5	0.17 (0.12, 0.23)	7	−0.08 (−0.14, 0.00)
		6	0.16 (0.11, 0.20)	7	−0.07 (−0.13, −0.01)
	*t*_max_, min	5	92 (75, 117)	6	45 (13, 80)
				7	74 (23, 164)
Peptide YY	AOB, pg L^−1^ min^−1^	1	173 (131, 225)	5	−80 (−134, −12)
				7	−133 (−199, −78)
		5	94 (43, 132)	7	−56 (−110, 0)
		6	146 (93, 187)	7	−108 (−177, −53)
	*A*_max_, pg mL^−1^	1	0.3 (0.2, 0.5)	7	−0.2 (−0.4, 0.0)
		5	0.3 (0.2, 0.5)	7	−0.2 (−0.4, 0.0)
		6	0.3 (0.2, 0.4)	7	−0.2 (−0.4, 0.0)
	*t*_max_, min	1	233 (152, 292)	5	−108 (−187, −29)
		5	125 (87, 166)	6	103 (21, 157)
**Metabolite**					
Betahydroxybutyrate	AOB, μmol L^−1^ min^−1^	5	131 (88, 184)	7	−86 (−153, −17)
	*A*_max_, μmoL L^−1^	5	0.3 (0.2, 0.5)	7	−0.2 (−0.4, 0.0)
Glucose	*t*_max_, min	1	123 (92, 150)	7	221 (11, 439)
Triglycerides	AOB, mmol L^−1^ min^−1^	5	61 (34, 85)	7	−40 (−65, −5)
	*A*_max_, mmol L^−1^	1	0.3 (0.1, 0.4)	7	−0.2 (−0.4, −0.1)
		5	0.3 (0.2, 0.4)	7	−0.3 (−0.4, −0.1)
		6	0.2 (0.1, 0.3)	7	−0.2 (−0.3, 0.0)
	*t*_max_, min	1	205 (184, 224)	5	−23 (−42, 0)
		5	181 (162, 201)	6	−27 (4, 47)
**Sensation**					
Hunger	AOB, min	1	−14 (−84, 53)	6	104 (14, 206)
		5	−92 (−26, 39)	6	114 (25, 224)
	*t*_max_, min	6	263 (13, 1183)	7	−113 (−684, −3)

a Values are means (95% CI) and correspond to the first of the two LEs under comparison.

b Values are means (HPD 95% CI) and correspond to the difference of the second LE compared to the first LE.

### 3.1. Gastric fat emptying

There were no differences in fat emptying between the four LEs. Only MC-LE6 had a lower initial postprandial fat volume (*V*_0_ of MC-LE6: 5% lower than PS-LE1) indicating that more fat emptied directly after meal intake. In terms of secretion, longer secretion emptying times were found for MC-LE6, with *T* of MC-LE6 49% longer than WPI-LE5 and 34% longer than CNC-LE7, suggesting that MC-LE6 was retained longer in the stomach. CNC-LE7 reached its minimum in gallbladder volume later than the other LEs, with *t*_max_ of CNC-LE7 40% longer than WPI-LE5, 44% longer than MC-LE6.

### 3.2. GI hormones and metabolites

**Fig. 3:**
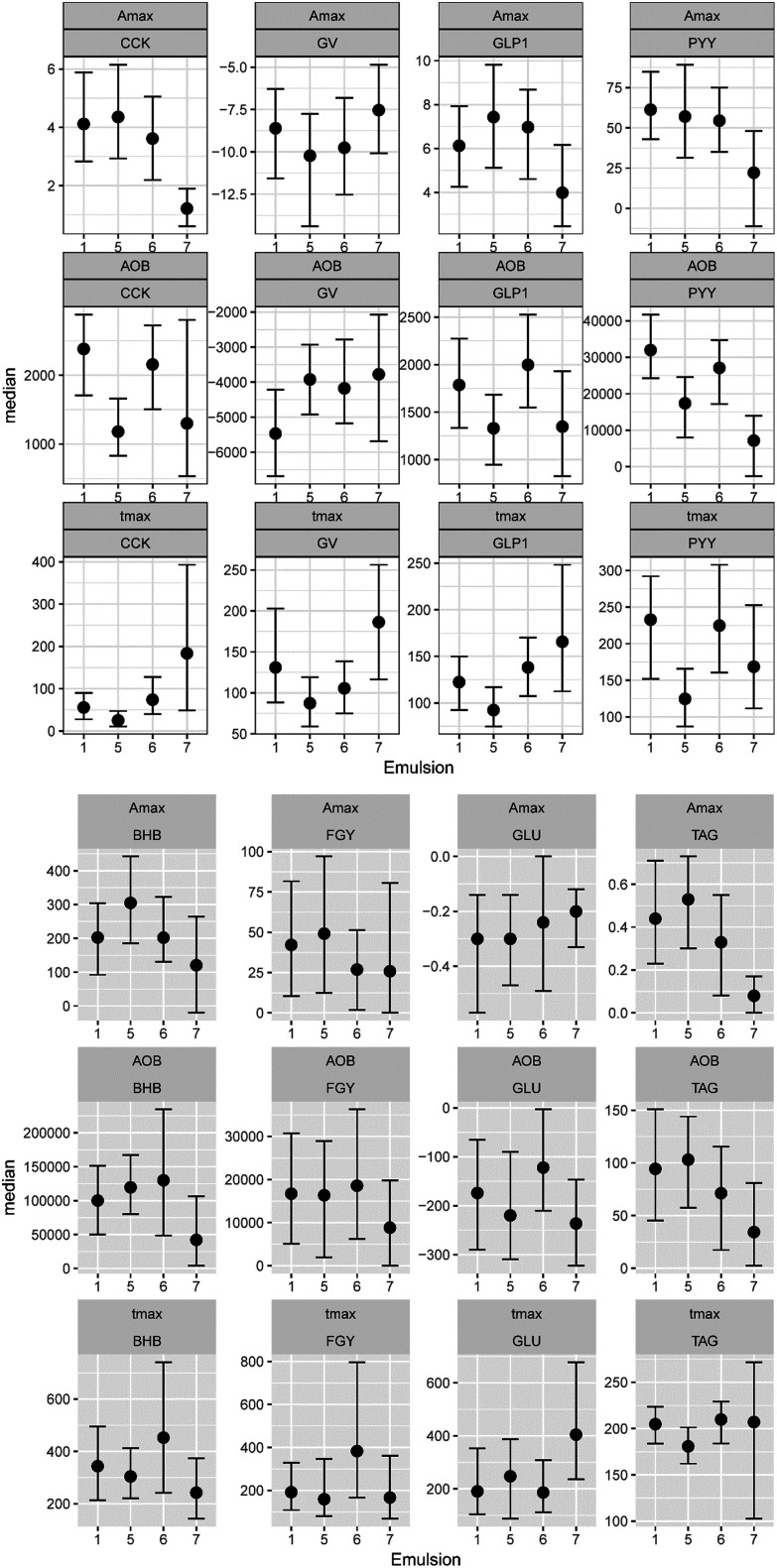
Boxplots displaying median and HPD 95% CI of the extracted parameters from the GI hormone and metabolite concentration curves in healthy human adults. Values are given for CCK in pmol L^−1^, GLP-1 in pg mL^−1^, PYY in pg mL^−1^, BHB in μmol L^−1^, GLU in mmol L^−1^ and TAG in mmol L^−1^.

**Fig. 4:**
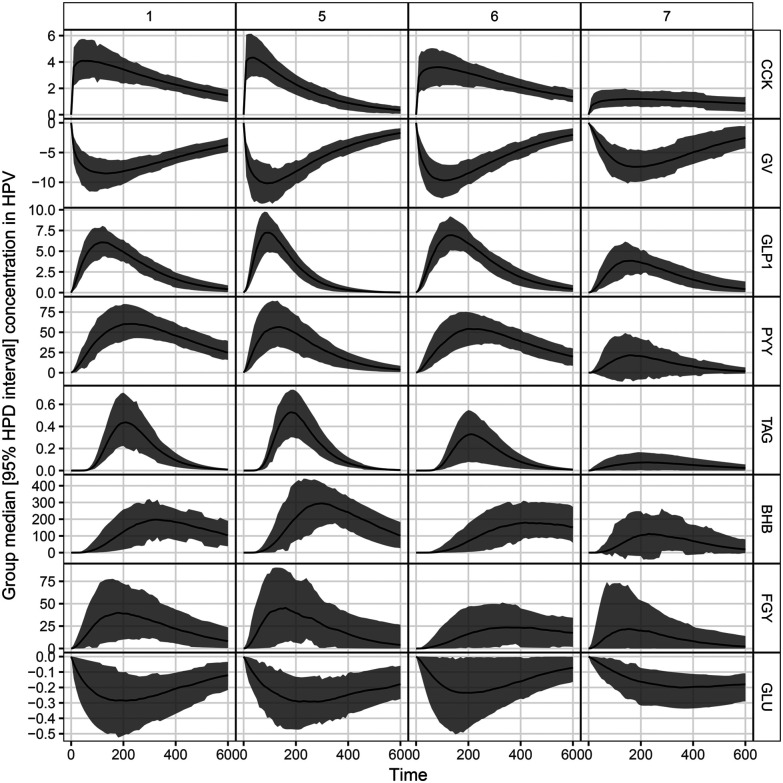
Group median DOB curves (black line) of GI hormone and metabolite concentrations with HPD 95% CI (gray area) in healthy adults over time (min) after LE consumption. The data is grouped by emulsion (columns). Values are given for CCK in pmol L^−1^, GLP-1 in pg mL^−1^, PYY in pg mL^−1^, BHB in μmol L^−1^, GLU in mmol L^−1^ and TAG in mmol L^−1^.

In Fig. 3, the complete set of parameter estimates AOB, *A*_max_ and *t*_max_ is visualized for each LE, grouped by hormone and metabolite. The group median curves for each LE and parameter are displayed in Fig. 4.

CCK profiles of CNC-LE7 demonstrated approximately 70% lower maximum response *A*_max_ compared to all other LEs. Effects of WPI-LE5 on CCK gave approximately 50% lower overall response (AOB) values compared to PS-LE1 and MC-LE6 and an earlier maximum response (*t*_max_ of WPI-LE5: 64% earlier than MC-LE6, 85% earlier than CNC-LE7). Emulsion effects on GLP-1 profiles were similar to, but less pronounced than those observed for CCK (*A*_max_ of CNC-LE7: 47% lower than WPI-LE5, 44% lower than MC-LE6; AOB of WPI-LE5: 34% lower than MC-LE6; *t*_max_ of WPI-LE5: 64% earlier than MC-LE6, 45% earlier than CNC-LE7). Comparable emulsion effects were detected also for PYY profiles. The AOB and *A*_max_ of PYY for CNC-LE7 demonstrated 60 to 77% lower responses compared to all other emulsions. The effects of WPI-LE5 on PYY gave approximately 46% lower overall response compared to PS-LE1 and likewise earlier maximum response compared to PS-LE1 and MC-LE6.

Emulsion effects on the metabolites were predominantly detected in the TAG response. The maximum TAG response for CNC-LE7 was distinctly lower compared to all other emulsions (*A*_max_ of CNC-LE7: 67% lower than PS-LE1, 100% lower than WPI-LE5, 100% lower than MC-LE6). Moreover, the maximum TAG for WPI-LE5 was reached earlier (*t*_max_ of WPI-LE5: 11% longer than PS-LE1, 18% longer than MC-LE6). An emulsion effect for CNC-LE7 was also detected in the BHB response with an approximately 70% lower maximum and overall response compared to WPI-LE5. CNC-LE7 affected GLU only by delaying the minimum response compared to PS-LE1.

### 3.3. Sensations

**Fig. 5:**
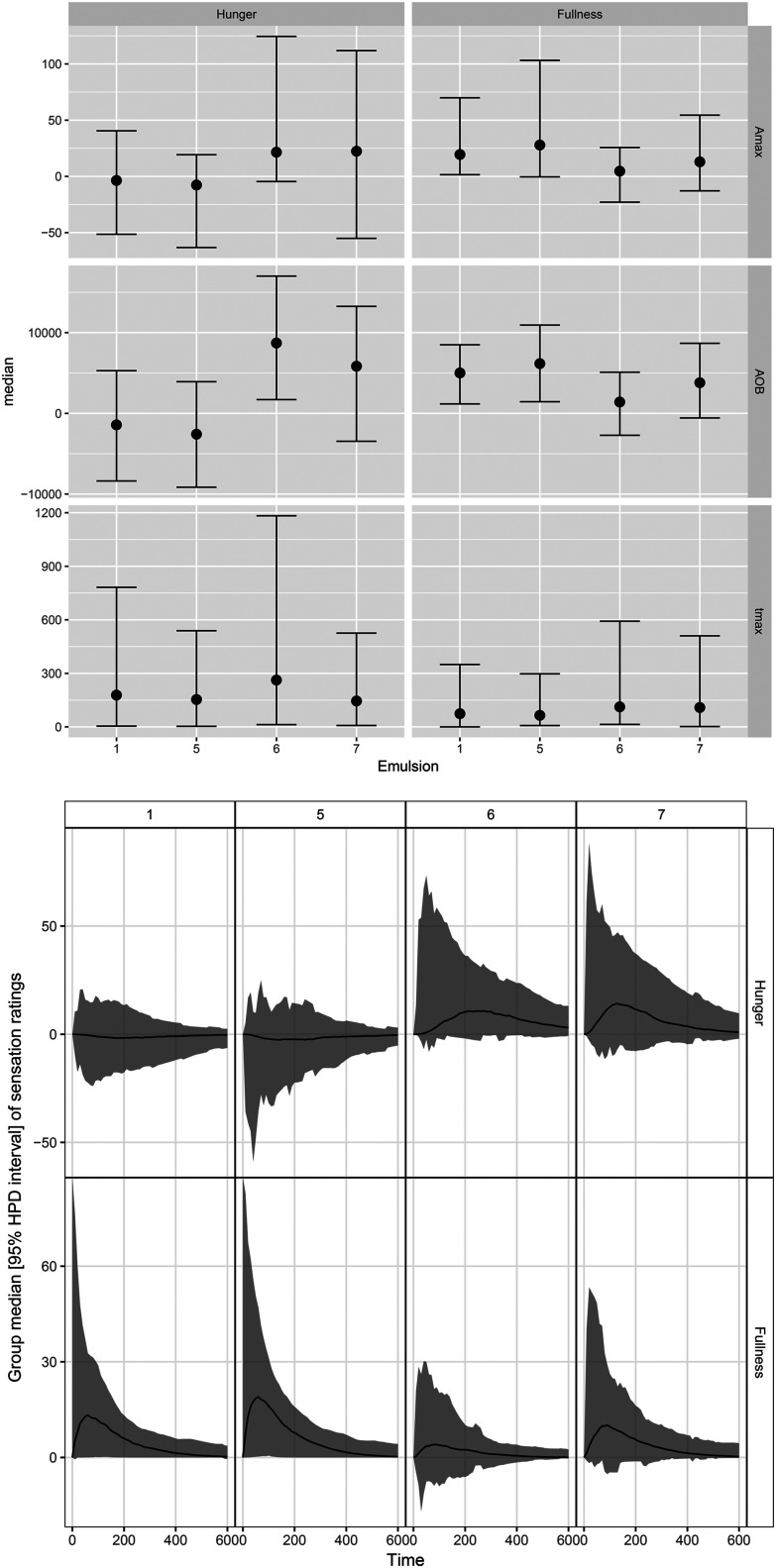
Top: Boxplots displaying median and HPD 95% CI of the extracted parameters from the reported hunger and fullness. Bottom: Group median DOB curves (black line) with HPD 95% CI (gray area) of hunger and fullness sensation ratings over time (min) after consumption of different LEs.

The median (95% HPD) visceral sensation ratings after consumption of different LEs are displayed in Fig. 5. Different dynamics in hunger/fullness perception were reported for PS-LE1 and WPI-LE5 compared to MC-LE6 and CNC-LE7. No hunger (no deviation from baseline) was reported for PS-LE1 and WPI-LE5, whereas hunger was initially perceived after consumption of MC-LE6 and CNC-LE7. In turn, more fullness was perceived for PS-LE1 and WPI-LE5 compared to MC-LE6. A similar trend was observed compared to CNC-LE7 but differences were not significant. Time to maximum hunger was more than twice as long for MC-LE6 compared to CNC-LE7. No other emulsion effects were detected. No negative visceral sensations (nausea, bloating, or epigastric pain) were reported by the subjects included in the study.

### 3.4. Food consumption

Table 2 displays the linear model estimates of food consumption 5 h after LE consumption. There was a main effect of sex on the amount of energy consumed. Whereas female subjects consumed in total 555 kcal (352, 748 kcal) on average, male subjects consumed on average 632 kcal (374, 1026 kcal). No emulsion effect was detected. However, there was an interaction between emulsion and sex. Male subjects ate less in total after MC-LE6 than after PS-LE1, 390 kcal (103, 670 kcal). This was mainly caused by a lower fat and protein consumption.

**Table 2:** Linear model estimates of food consumption from the *ad libitum* buffet in healthy subjects. Values are means (95% CI)

	Total, kcal	Fat, kcal	Carbohydrates, kcal	Protein, kcal
Intercept^a^	555 (352, 748)	184 (88, 291)	286 (204, 370)	85 (46, 129)
Buffet time, h	75 (−14, 167)	33 (−7, 72)	31 (−17, 75)	16 (−1, 32)
**Emulsion**				
E5	123 (−35, 288)	81 (5, 154)	27 (−62, 119)	13 (−18, 43)
E6	97 (−77, 274)	68 (−8, 145)	7 (−78, 94)	25 (−6, 55)
E7	41 (−137, 214)	19 (−67, 108)	18 (−78, 113)	9 (−21, 42)
**Gender**				
Male	632 (313, 982)	280 (110, 438)	229 (94, 363)	117 (43, 184)
**Emulsion × gender**				
E5 × men	−99 (−357, 169)	−91 (−206, 41)	19 (−129, 163)	−27 (−73, 17)
E6 × men	−390 (−670, −103)	−208 (−332, −87)	−89 (−231, 51)	−99 (−146, −48)
E7 × men	−188 (−494, 134)	−90 (−231, 50)	−39 (−200, 129)	−62 (−120, −6)

a PS-LE1, women, 12 pm buffet time.

## 4. Discussion

### 4.1. Effect of LE interfacial design on gastric emptying and satiation hormone response in humans

The four different interfacial stabilizers had distinct effects on *in vivo* digestion in humans. MC-LE6 exhibited a different pattern of gastric fat emptying compared to the other LEs. This is likely due to the stable and even fat distribution of MC-LE6 in the stomach as previously observed using MRI.^7^ In contrast, the protein-stabilized WPI-LE5 coalesces and creams in the stomach whereas the particle-stabilized CNC-LE7 demonstrates a strong gastric gelling. MC-LE6 initially exhibited faster fat emptying. After this initial phase the fat emptying rate began to slow down and the secretion emptying times were longer than for other LEs. The initial faster fat emptying of MC-LE6 may trigger mechanical (stomach distension) and biochemical (earlier fat sensing) cues that down-regulate gastric emptying leading to a slower late phase emptying. An alternative explanation could be due to the higher extensional viscosity of MC-LE6 discussed below. Previous MRI studies found that overall gastric emptying can be delayed for gastric-unstable LEs due to lipid coalescence and creaming in the stomach.^13,14,39^ We did not observe this effect here for the protein-stabilized WPI-LE5. However, these studies measured gastric meal including water and fat, and emptying was measured indirectly *via* either breath testing or *via* MR spectroscopy in local volumes of stomach content. A direct comparison with these results is therefore difficult. It is further possible that the change between sitting and lying positions between MRI measurements decreased the effect of fat layering.^40^ Ultimately, it is possible that inter-individual differences in fat emptying rates may be larger than the inter-emulsion differences.

The satiation hormone responses were assessed with reference to CCK, PYY, and GLP-1. Responses were fastest and highest for the protein-stabilized WPI-LE5. This is likely because the proteins are not able to prevent lipolysis as they are rapidly hydrolyzed by gastric proteases and readily displaced by gastric lipases.^19,20^ The fast hormone response for WPI-LE5 could at first seem contradictory considering the lipid coalescence and creaming observed in the stomach that drastically reduces the interfacial area. Previous studies found increased CCK release for gastric-stable compared to gastric-unstable LEs.^13^ However, liquid fat is readily redispersed by stomach movements which increases the specific surface area upon emptying into the duodenum.^25,26^ Despite exhibiting the highest GI hormone response, the overall hormone response (AOB) was lowest for WPI-LE5, which could be associated with a less sustained perception of satiety. MC-LE6 demonstrated a lower and delayed hormonal response compared to reference PS-LE1 despite comparable gastric structuring, potentially due to the thermo-gelling of MC at human body temperature that prevents lipase adsorption.^31–33^ Interestingly, CNC-LE7 demonstrated a drastically reduced satiation hormone and metabolite response when compared to other LEs. Further, the minimum gallbladder volume was also reached later for CNC-LE7. Collectively, this suggests a constrained amount of fat sensing with CNC-LE7. This is in agreement with *in vitro* digestion experiments which reported that particle-stabilized (Pickering) LEs are efficient at preventing lipase adsorption.^7,10,21,41^ An additional explanation could be related to the strong gastric gelling previously observed for CNC-LE7 using MRI.^7^ CNCs were also found to reduce lipolysis when present in food other than LEs.^42^ The limited lipolysis suggest that Pickering stabilized emulsions such as CNC-LE7 can efficiently protect sensitive lipophilic nutrients and drugs from the harsh gastric environment and could be of interest as oral delivery systems.^3,18^

The delivery of undigested nutrients to the ileum has often been associated with activation of the “ileal brake”, which triggers satiation, reduces hunger, and delays further gastric emptying.^43^ We do not observe an ileal brake here despite using two emulsions (MC-LE6 and CNC-LE7) which efficiently prevent gastric lipolysis and therefore deliver undigested lipids to the ileum. On the contrary, we find no considerable variations in fat emptying rates and even a decreased GI hormone response for LEs that deliver undigested lipids to the ileum. Hence, our results suggest that the accessibility of lipids for lipases and rate of lipolysis determine satiation rather than the state of delivered lipids.

### 4.2. Effect on hunger sensation and food intake

Subjects reported no hunger and more fullness after consumption of PS-LE1 and WPI-LE5. This is in agreement with the faster and higher plasma GI hormone and metabolite response observed for PS-LE1 and WPI-LE5, confirming that the chosen GI hormones are good proxies for hunger/fullness. In contrast, more hunger and less fullness were reported after consumption of MC-LE6 and CNC-LE7. Subjects further reported delayed hunger perception after consumption of MC-LE6. The larger accumulation of secretion and the resulting larger stomach distension could be possible explanations. These results demonstrate that LE interfacial design has a direct effect on satiation beyond gastric stability, *i.e.* LEs that are able to delay lipolysis such as MC-LE6 and CNC-LE7 delay satiation.

### 4.3. Comparison to *in vitro* lipolysis and potential animal models

*In vivo* validations of LE digestion remain an important step towards the development of functional LEs with the potential to alter digestion and satiation response. In recent years, the continuous optimization of *in vitro* digestion models has facilitated a better prediction of *in vivo* LE digestion.^23^ Nevertheless, we observe certain discrepancies here compared to our previous *in vitro* digestion experiments of the novel LEs.^7^ For example, we observed faster *in vitro* lipolysis for MC-LE6 compared to WPI-LE5. This is likely because MC-LE6 has a smaller droplet size and is evenly distributed in the simulated gastric juice. Here, we observed a delayed satiation response for MC-LE6 indicating slower lipolysis. Hence, *in vitro* protocols may fail to mimic physiological digestive processes like gastric structuring and mixing. It is important to note that differences in *in vitro* gastric digestion between LEs were only observed after mixing with simulated gastric fluid and addition of gastric mucin that induced different LE structuring, which is currently not part of the INFOGEST protocol.^7^ Furthermore, we here observed effects of LE interfacial design on gallbladder secretion and fat emptying rates. For such physiological responses to specific meals *in vivo* testing remains crucial. However, we have recently demonstrated that rats exhibit a similar LE gastric structuring, emptying, and GI hormone response as humans,^24^ and rats thus present a promising animal model for human LE digestion.

### 4.4. Comparison to previously investigated LEs (LE1–4)

In comparison to reference PS-LE1 (surfactant-stabilized, gastric stable, small droplets), a faster fat emptying and GI hormone response was typically observed for gastric unstable LE4 stabilized by a mixture of proteins (caseinate) and monoglyceride.^12,24,28,29^ We did not observe faster fat emptying here for protein-stabilized WPI-LE5 despite fast phase separation and fat layering in the stomach typical for protein-stabilized LEs.^7^ Nevertheless, WPI-LE5 induced a characteristically fast and high GI hormone response compared to current and previously tested LEs, underlining the fast degradation of proteins by proteases and/or displacement by lipases. The fast GI hormone response to WPI-LE5 despite fat layering is due to the re-dispersion of lipid upon emptying into the duodenum. This effect has been previously studied using LE3, which has an equal emulsifier composition as LE4 but contains a fraction of solid fat that cannot be re-dispersed. This results in the formation of large fat aggregates and a delayed GI hormone response due to a decreased surface area for lipolysis.^24–27,29^ Therefore, LE3 has been associated with a characteristically low fullness and high hunger perception.^26,29^ Conversely, gastric stable LEs with initially large droplet size (LE2) show a less pronounced effect as the liquid fat can be re-dispersed.^26,29^ Compared to the previously investigated LEs, the newly designed MC-LE6 and CNC-LE7 provide a new approach towards modulating LE digestion and satiation response *via* LE interfacial design. MC-LE6 exhibits a similar gastric mixing as surfactant stabilized PS-LE1,^7,12^ but a reduced GI hormone response. Thus, MC appears more effective at preventing lipase adsorption. This may be due to the thermo-gelling of MC at human body temperature.^31–33^ Finally, CNC-LE7 is the first LE system investigated *in vivo* stabilized by a Pickering mechanism. Strikingly, we observed a significant reduction in plasma GI hormone and metabolite response compared to all other investigated LEs. Despite the increasing interest in Pickering emulsions, this is to our knowledge the first report of *in vivo* Pickering emulsion digestion and satiation hormone response in humans along with our preliminary report.^7^

### 4.5. Future considerations: effect of extensional viscosity and oil type?

**Fig. 6:**
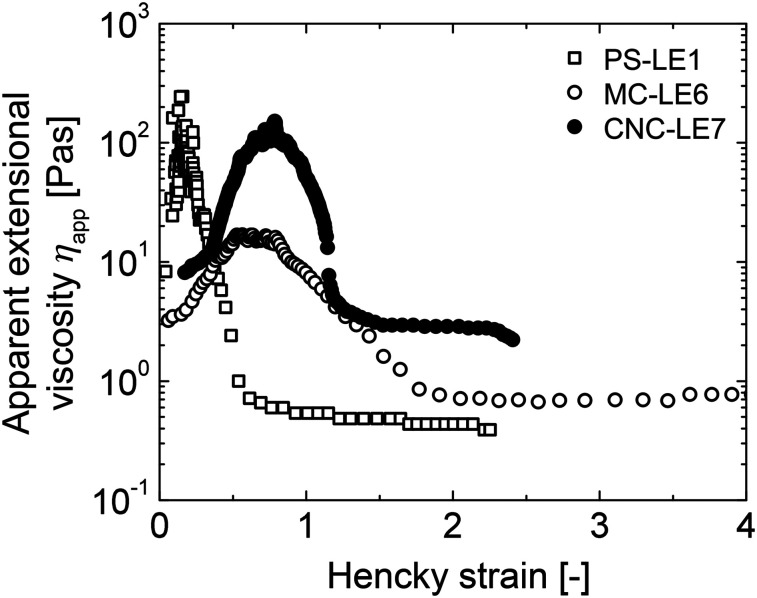
Apparent extensional viscosity of PS-LE1, MC-LE6, and CNC-LE7 as a function of Hencky strain.

Meal viscosity can play an important role in the digestion and satiation response.^44^ Here we propose a combined approach of utilising the shear viscosity and extensional viscosity to obtain the rheological fingerprint of the LEs during stomach flow and, in particular, during secretion emptying. Although the shear viscosity was in a similar range for all four LEs, the actual consistency still differed particularly between PS-LE1 and MC-LE6, which can be attributed to the extensional viscosity (see Fig. 6). While PS-LE1 shows a narrow peak in apparent extensional viscosity indicating a rapid flow alignment of the emulsion, the extensional viscosity of MC-LE6 and CNC-LE7 are observed in a wider Hencky strain regime. Both LEs demonstrate a strain hardening behavior, *i.e.* more resistance to elongation flow due to structural rearrangement, alignment, and orientation in flow direction. This was confirmed in practice as it was more difficult to pass MC-LE6 through a syringe during meal infusion. As MC-LE6 has longer secretion emptying time and a triggered a distinctively high gallbladder secretion compared to other LEs, we speculate that these differences may be induced by the higher extensional viscosity of MC-LE6 under gastric conditions, *i.e.* it is harder for the stomach to push a strain hardening fluid through the pylorus. This finding indicates that not only the emulsion stabilization by different interfacial active components and their interaction with gastric fluids but also the resulting LE structures and their complex rheological properties influence food digestion.

The model LEs are commonly prepared with canola oil. However, the adsorption behavior and interfacial structure of emulsifiers, *i.e.* surfactants,^45^ proteins,^46^ and nanoparticles such as CNCs^47^ strongly depend on the polarity of the used oil. There have already been reports of altered *in vitro* digestibility of emulsions stabilized with proteins^20,48^ and CNCs^10^ depending on the used oil. We therefore assume that the oil chemistry could also influence the *in vivo* digestion of LEs, and altering oil types could be considered for future LE digestion experiments.

## 5. Conclusions

Lipid emulsions (LEs) with tailored digestibility have been recognized for satiation control and the delivery of drugs or nutrients, however, *in vivo* validations of altered LE digestibility in humans remain scarce. We have formulated three novel LEs with custom interfacial designs to unravel their potential to modulate human *in vivo* digestion and satiation. Namely, we investigated LEs stabilized by whey protein isolate (WPI-LE5), thermo-gelling methylcellulose (MC-LE6), and solid cellulose nanocrystals (Pickering CNC-LE7) in comparison to the widely studied surfactant-stabilized PS-LE1. We recognized two main effects of LE interfacial design on *in vivo* digestion modulation. Firstly, magnetic resonance imaging revealed that LE interfacial design dictates gastric structuring. WPI-LE5 showed gastric coalescence and creaming typical for protein-stabilized LEs, MC-LE6 was evenly dispersed in the stomach similar to PS-LE1, while CNC-LE7 showed strong gastric gelling. Secondly, satiation response in terms of plasma metabolites and satiation hormones varied significantly following consumption of different LEs depending on their ability to prevent lipase adsorption. WPI-LE5 induced a fast and high metabolite and hormone response due to fast hydrolysis and displacement of proteins, while the thermo-gelling methylcellulose was more effective at preventing lipase adsorption and reduce satiation response. Most notably, CNC-LE7 only induced a minor satiation response, underlining the potential of Pickering LEs to prevent lipase adsorption, limit physiological satiation responses, and deliver sensitive lipophilic compounds. While the effectiveness of Pickering CNC-LE7 to prevent lipolysis was predicted by *in vitro* experiments, the ability of MC-LE6 to prevent lipolysis was underestimated *in vitro*. Hence, despite the steady improvement of *in vitro* digestion protocols, *in vivo* validations remain critical to understand the behavior of LEs under physiological conditions and their potential to modulate satiation.

## Conflicts of interest

There are no conflicts to declare.

## Supplementary Material
